# Does Vagal Nerve Activity Predict Performance in a Naval Commando Selection Test?

**DOI:** 10.1007/s10484-025-09702-4

**Published:** 2025-03-20

**Authors:** Yosef Kula, Zev Iversen, Adi Cohen, Ariel D. Levine, Yori Gidron

**Affiliations:** 1 Department of Nursing, Faculty of social welfare and health sciences university of Haifa, Haifa, Israel; 2 Behavioral Science Branch, Israeli Navy, IDF, Haifa, Israel; 3 The Israel Naval Medical Institute, Haifa, Israel; 4 Bnai Zion Medical Center Israel, Haifa, Israel

**Keywords:** Selection procedures, Heart rate variability, Vagal nerve, Military performance, Stressful environment

## Abstract

Special operations forces (SOF) soldiers are elite fighters and tactical professionals who perform in high-stress environments. SOF selection processes aim to identify candidates who can sustain performance in high-stress and changing conditions. The vagal nerve is a crucial moderator of stress responses, and its activity (indexed by heart rate variability, HRV) has been shown to predict performance and psycho-physiological resilience in various settings. However, its predictive validity needs to be clarified. This study examined the relationship between HRV and success in an intensive selection procedure. In a historical prospective study, we derived an HRV parameter (root mean square of successive differences between normal heartbeats, RMSSD) from a 10-second ECG of 365 candidates for an SOF naval unit. The ECG was taken approximately two months before the selection procedure. The predictive validity of other routinely obtained measures was also considered. High RMSSD was significantly associated with success, but this relation disappeared after controlling for confounders (e.g., running score). However, after matching pairs of successful and non-successful candidates on confounders, HRV was again significantly related to course performance. The results of this study support the predictive value of HRV for tactical professionals. Given the high cost of training elite soldiers and the burden they undergo, improving accuracy of the selection processes may reduce the burden on candidates and lead to resource savings. Future studies should measure HRV at several time points with longer ECG records.

## Introduction

Tactical professionals (e.g., military and law enforcement) are required to maintain high physical fitness and specific technical skills (Scofield & Kardouni, 2015). Their working environment is defined by multiple stressors that may challenge their autonomic nervous systems (ANS). The unique requirements, alongside complex training processes, make the selection of suitable candidates for tactical positions critical and challenging (Sharp et al., 2012). Recent works demonstrate that ANS activity monitoring can be used to indicate physical fitness and performance in tactical settings (For a review, see Stephenson et al., 2021; Tomes et al., 2020). The current study will examine the ability of vagal activity measures to predict performance in an intensive commando selection test.

Special operations forces operators (SOF) are elite fighters who perform unique tasks requiring advanced physical and cognitive abilities. SOF work and training are defined with psychological and psychological stressors, so SOF soldiers are required to be flexible and adaptive to changing environmental conditions (Sharp et al., 2012). Since the ability to function under stress is critical for SOF soldiers, these units’ selection and training processes aim to identify the most suitable candidates to sustain performance and recover in high-stress environments. Finding objective measures that can assess individuals’ resilience when facing stress could have implications for many fields that require performance under stressful conditions (Picano et al., 2017).

The concept of stress is used in the literature in different ways and may describe a stimulus, a response to the stimulus, and/or the physiological consequences of the response (Kemeny, 2003). This article refers to stress as any physiological or psychological stimulus that requires an adaptive response from the individual (Byron et al., 2010; Vartanian et al., 2022). A related concept is allostasis (McEwen, 2003), which reflects the body’s wear and tear from chronic stress, and the current study’s context is more of a long-term acute stressor.

The way the individual perceives the stimulus affects the stress response. When an individual perceives his or her resources as insufficient to cope with, the situation may be evaluated as a threat, and a chain of physiological reactions begins, described as the stress response (Lazarus & Folkman, 1984; Folkman et al., 1986; Tomaka et al., 1993). Empirically supporting the importance of appraisal, Harvey et al. (2010) demonstrated that only when an individual perceived the situation as a threat there is a positive correlation between demands/ resources and cortisol. Within the military setting, stress stimulation can impair various aspects of performance, such as memory and decision-making (e.g., Delgado-Moreno et al., 2017; Gamble et al., 2018; Tornero-Aguilera & Clemente-Suárez,^2018^).

Responses and adaptation to stress are influenced by several body systems, including the ANS, which comprises the sympathetic and parasympathetic nerve systems (SNS and PNS) (Godoy et al., 2018a, b; Gunnar & Quevedo, 2007). When a stimulus is classified as a threat, the SNS begins a rapid response (within seconds) to release catecholamines (neurotransmitters), which causes, among other things, an increase in heart rate (HR) and breathing rates. (Kemeny et al., 2003). Alongside the activation of the sympathetic nervous system, there is an activation of the hypothalamus-pituitary-adrenal axis (HPA) (Kemeny et al., 2003; Russell & Lightman, 2019). HPA activation leads to the release of cortisol, impacting brain regions involved in cognitive processes, such as the frontal cortex, the hippocampus, and the amygdala (Charmandari et al., 2005; Kemeny et al., 2003).

Identifying factors that predict candidates’ resilience to stress and combat performance has been a consistent focus of both military research and other fields (e.g., Bartone, 1999; Bartone et al., 2008; Pyne et al., 2016). Identification and pre-selection of resilient candidates may lead to strengthening and improving the coping ability of the individuals and units (Iversen, 2018). Resilience describes the individual’s ability to adapt to stress and even grow in adversity (Vella & Pai, 2019; Wald et al., 2006). The main difference between definitions of resilience is the conceptualization of resilience as a personality trait versus a dynamic process (Herrman et al., 2011). Cognitive resilience, as defined by Stall and others (2008), is the “capacity to overcome the negative effects of setbacks and the associated stress on cognitive function or performance” (Staal et al., 2008, p. 260). Within the SOF context, cognitive resilience is critical because operators are required to function in extreme conditions (Flood & Keegan, 2022; Hormeño-Holgado et al., 2019). There is some evidence that Resilience is positively correlated with performance in various settings (e.g., health care and military settings). Handini et al. (2020) demonstrated a positive correlation between resilience and nurses’ performance in 11 cross-sectional studies. In the military setting, a recent systematic found evidence of a positive association between resilience and performance in weapon operators’ courses (5 studies), captivity training (2 studies), and diving training (1 study) (Jones et al., 2022).

The vagus nerve, known as the 10th cranial nerve, is the main nerve of the PNS branch of the ANS. Its activity moderates the intensity and duration of the stress response, as reflected in blood pressure, cortisol levels, and inflammation (Weber et al., 2010). The vagus nerve begins in the brainstem’s medulla and reaches the viscera. The nerve is divided into sensory branches (80%) and motor branches (20%) (Hermanowicz, 2007). The sensory branches collect and transmit sensory information, including information about blood pressure, pain, temperature, and inflammation, and translate it into commands for action through the motor branches of the vagal nerve (Câmara & Griessenauer, 2015; Circuit, 2006; George et al., 2000). The ANS (PNS and SNS) and the endocrine systems are connected via the hypothalamus (Mueller et al.,^2022^). On the afferent side, vagal projections are conveyed to the hypothalamus through adrenergic and noradrenergic projections and directly trigger the HPA axis (Swanson & Kuypers, 1980). On the efferent side, the hypothalamus and the remaining slow glands of the HPA axis can trigger SNS activity (Mueller et al.,^2022^).

The PNS activity modulates heart rate, which slows down the sinus pacemaker (Shaffer et al., 2014). The response time and duration of the PNS effects on heart rate are faster and shorter than those of the SNS (Nunan et al., 2010). The PNS speed and predominance mean that heart rate changes at rest mainly reflect PNS vagal activity (Berntson et al., 1997). Heart rate variability (HRV) is a measure that represents the changes in the heart rate interval between one heartbeat and another and can be used to assess and monitor vagal nerve activity (Kuo et al. 2005; Sammito et al., 2016; Shaffer et al., 2014). Many studies use the assessment of vagal activity by HRV at rest (Tonic HRV) as a measure of self-regulation ability and adaptation to different challenges (Spangler & McGinley, 2020). Measuring HRV during activity or recovery provides information on how the individual responds to and recovers from stressful conditions. When interpreting HRV data, the time point and context in which the measurement is performed must be considered a function of the expected physiological response. For example, during rest, high HRV is considered adaptive in most cases (Laborde et al., 2017).

The screening and selection processes for commando units are designed to identify the candidates who will be able to maintain performance under intense pressure (resilient candidates) (Sharp et al., 2012). In order to identify those candidates, the screening procedures for SOF examine various aspects of functioning under psychological stressors (e.g., time pressure) and physiological (e.g., sleep deprivation and cold water) (Ledford et al., 2020; Lieberman et al., 2002; Smith et al., 2020). To function under these conditions, applicants must have extreme physical and mental capabilities and rapid adaptive responses to changing environments. Those capabilities were found to be related to vagal activity, as represented by HRV. High HRV enables a moderate physiological response and faster recovery from acute stress (Weber et al., 2010), reflecting better resilience. In a recent systematic review, High HRV was correlated with cognitive flexibility and inhibition, crucial elements of functioning under stress and executive functioning (Magnon et al., 2022). In addition, HRV positively correlated with brain activity in frontal regions, including the anterior cingulate cortex and the ventromedial prefrontal cortex, which are essential for attention switching (Andrewes & Jenkins, 2019; Gitelman et al., 1999). All these neurophysiological capacities are essential for performance under severe stressful environments and a challenging selection test.

Several studies have examined the relationship between HRV and performance in intensive military training. In a study that included three prospective studies in intensive US Army military training (survival training and combat diving training), low HRV just before training predicted better performance during training. It is possible that the measurement of HRV took place just before exposure to a severe stressor, which soldiers anticipated. Therefore, those who demonstrated a lower HRV (i.e., showed an SNS response) had a more adaptive response to the already stressful context. the measurement of HRV just before a challenging event may not represent “true” resting conditions (Morgan et al., 2007). Another study supported this explanation in which HRV was measured several months before intensive and highly stressful training (“hell week”). Indeed, in that study, it was found that participants with high HRV had a higher chance of passing the training (Stanfill, 2012). As mentioned above, the context of measuring HRV is crucial for his predictive role (Laborde et al., 2017; Vincent et al., 2021).

The predictive value of HRV in relation to performance in extremely stressful military conditions is unclear since, as cited above, there is inconsistency in the literature. The present study thus aimed to examine the ability of resting HRV to predict performance (success) in a highly stressful environment such as a naval commando selection course. Since HRV was measured approx. two months before the stressful selection test, thus representing resting HRV, we hypothesized that participants with higher levels of HRV would pass the test at a greater rate than those with low levels of HRV. These differences were expected to remain after controlling for the effects of other background factors and predictors.

## Methods and Materials

### Participants and Procedure

**Fig. 1:**
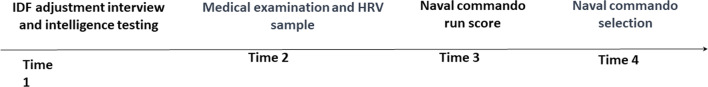
The selction process for the commando units

Participants were past candidates for naval commando training. In Israel, military serviceis mandatory at age 18, and selection processes for elite units are conducted during high school prior to military requirement (see Fig. 1).

Candidates found to be suitable after a psychological evaluation and medical tests are invited to a 5-day selection course that includes physical and mental stressors and is characterized by high attrition rates. The five-day course is held several times yearly, and the medical examinations (including the ECG) are carried out about two months before the selection test. Candidates who successfully pass the selection course begin training after high school. Like other militaries, the Israel Defense Forces (IDF) do not share data regarding success rates. However, it can be said that the percentage of candidates who pass the selection test is between 10 and 20%. In the present study, the participants were 356 healthy high school male students aged 16–17 who were candidates for the naval commando training. We arrived at this final number after excluding cases with unreadable ECG (due to military confidentiality issues, we cannot provide the initial number of cases).

For calculating the required sample size prior to the study, two calculations were made: first, assuming a correlation of 0.2 between HRV and success at the selection course, with a statistical significance level of 0.05 and a statistical power of 0.80, 193 soldiers were needed (Cohen & Cohen,^1983^). Second, performing a t-test, in which the HRV levels of dropouts versus successful ones were compared, based on the data of Alalyan and others (2020), and assuming a 10–20% difference in HRV levels between successful and dropout cases, 108–403 soldiers were needed. An online calculator sample size calculation was used (www.stat.ubc.ca).

## Study Design

The study was retrospective (pseudo-prospective), and data from the candidate files were analyzed anonymously. HRV was derived from ECG in medical files from medical tests performed 2–3 months before the selection test (T1), which were used to predict the outcome of the candidate’s performance at the selection course later (T2). The ECG charts from the selected sample were manually scanned and analyzed using a method adopted in our laboratory and reported in other studies (Atar et al., 2023). The analysis of the ECG charts was done by the analyst, who was blind to the performance of the candidates. The data were analyzed approximately two years after the candidate’s participation, using data that is obtained routinely, and the use of the data had no influence on the candidate selection. The study was approved by the IRBs of the institutions that led it.

## Physiological Measures

HRV measures were taken from a standard 10-second ECG chart routinely obtained from all candidates approximately two months before the selection test (This was the only available ECG). To calculate HRV, we chose the index of route mean square of successive differences (RMSSD) since it is considered to represent vagal activity and is less influenced by respiratory activity (Thayer & Lane, 2000). RMSSD was found to be reliable for estimating HRV from short ECG charts (Nussinovitch et al., 2011). To measure RMSSD, we used the Adobe Acrobat software (www.adobe.com) to measure inter-beat-intervals as the basis of HRV manually. We obtained very high inter-rater reliability levels (*n* = 8, *r* = 0.9). RMSSD values are presented in milli-seconds (ms), and heart rate is presented in beats per minute (BPM).

## Fitness: The 2 km Run Score

2 km run: IDF converts run time into a score of 1-100, whereby a score of 100 represents the shortest possible run time and is indicative of high-level physical fitness. The conversion is performed according to a table the IDF Combat Fitness Corps developed, and the measurement was performed by IDF staff.

## Psychological and Cognitive Measures

### Adjustment Interview

All candidates undergo a personal interview examining their suitability to adapt successfully to military combat service. Interviews are conducted by soldiers who underwent rigorous selection processes and were trained specifically for this task in an intensive course. The adjustment interview is a structured interview developed by Daniel Kahneman in the 1950s and updated over the years by IDF Behavioral Science Center psychologists. It remains a centerpiece of IDF and assessment alongside intelligence testing. The structured interview assesses different personality components, a general impression of the interviewer, and a prediction of the ability to adapt to combat military service. The final score consists of a weighting of the personality and prediction components, with scores ranging from 8 to 40 (State Comptroller of Israel, 2016, 2023).

## Intelligence Testing

This test examines the candidate’s cognitive ability and consists of a series of cognitive tests, including verbal, quantitative, and spatial components. The score ranges from 10 to 90 in 10-point intervals (IDF, 2021; State Comptroller of Israel, 2023). IDF has used intelligence testing since the 1950s, the current version was developed by the IDF Behavioral Science Center.

## The Outcome of the Selection Course

This constituted our main dependent variable– pass or fail at selection. Evaluation at the selection course is conducted by a cadre of naval commando reserve personnel. Eight cadre members evaluate each candidate throughout the 5-day course. Assessment includes objective measures (run times, swim times, etc.) and subjective components (integrity, sociability, etc.). The naval commando training center commander makes the final decision based on scores and input from all eight cadre members.

### Statistical Analysis

We used a two-stage approach to analyze this study’s data. First, we analyzed the entire sample using *t*-tests to examine differences in continuous measures between candidates who passed and failed the selection test. Then, significant predictors and RMSSD (not log-transformed)were entered in a multivariate logistic regression using enter method. Second, as explained in more detail below, we focused on a smaller sample of paired candidates (pass versus fail), matched on specific confounders, and then examined differences in HRV between these groups with a paired *t*-test. Statistical analyses were conducted using IBM SPSS Statistics 27.0 software.‏ The significance level was set at *p* < 0.05 one-tailed.

## Results

All participants were between 16 and 17 years old, male, and in good health when undergoing the ECG and subsequently 17 years old when undergoing the navy selection test.

The mean HRV in those who later passed the selection test (*M* = 108.91, *SD* = 79.96) was significantly higher than those who failed (*M* = 83.77, *SD* = 58.94), *t*(354) = 2.13, *p* = 0.017). As in many other studies, the RMSSD data were non-normally distributed (Skewness = 1.88; Kurtosis = 4.77), hence, a log transformation was applied as recommended (Laborde et al., 2017). After the transformation, RMSSD was still significantly higher in those who passed the selection test *t*(354) = 1.77, *p* < 0.05. For comprehensibility, the means are presented without the transformation.

Additional t-tests were performed concerning the outcome of the selection test (see Table 1). High combat fitness, suitability to combat service, psycho-technical score, and low HR significantly predicted passing the selection test.

**Table 1:** t-test for selection test outcomes

	Pass		Fail				
	M	SD	M	SD	t (df)	*p*	Choen’s d
Combat fitness test	88.000	6.6939	75.305	11.3133	-5.94 (353)	< 0.001	1.15
Suitability to combat service	32.69	3.274	31.41	3.410	-1.938(354)	0.026	0.37
Psycho-technical test	75.86	12.961	70.03	12.930	-2.32(354)	< 0.011	0.45
RMSSD	108.91	79.96	83.77	58.94	2.13 (354)	*0.017*	0.41
Heart rate BPM	57.00	10.107	64.40	11.112	3.40(352)	< 0.001	0.67

All measures (heart rate, run-score, suitability for combat service measures, intelligence scores) were significantly higher among those who later passed the selection test. This was also observed using the nonparametric Mann Whitney U test *p* = 0.03 one-tailed.

### Logistic Regression

Logistic Regression analysis was used to evaluate the predictive value of HRV for success in the selection test alongside the other predictors mentioned above (HR was not included due to missing data). The run score was the only significant predictor, with Odds ratio (O.R) = 1.114, 95% CI: 1.084–1.208, *p* = 0.001. HRV no longer significantly predicted success.

### Matched Sample

Due to the low ratio of pass-to-fail typically seen in such selection tests and our sample, and to increase statistical power beyond the effect of cofounders, we decided to perform an exploratory analysis with a matched sample. Candidates were paired and matched on significant confounders: the 2 km run score and the adjustment interview score (the matching level was ± 5 points for both variables). In this analysis, a paired t-test was used. The mean heart rate variability (HRV) of individuals who passed the selection test (M = 108.91 ms, SD = 79.96) was significantly higher than that of those who failed (M = 74.97 ms, SD = 53.12), *t*(28) = 1 0.193, *p* = 0.019 (one-tailed); *p* = 0.38 (two-tailed) Cohen’s *d* = 0.4.

## Discussion

In the present study, the main finding is that initial HRV levels were significantly higher in candidates who later passed the selection test. This is in line with our hypothesis and with the findings of Stanfill et al. (2012). Our results also point out the crucial importance of the timing of measuring HRV. When HRV is obtained several months before a selection test, thus reflecting a “real” resting HRV, higher HRV should predict better performance. The research literature indicates that a high HRV at rest is beneficial in most cases (Thayer et al., 2012). When the measurement is taken just before a highly stressful situation, as in the work of Morgan et al. (2011), low HRV may represent a more adaptive SNS response to the stressor. In contrast, high HRV obtained in a calmer context may reflect a more “real” resting measure and represent better adaptivity, thus predict better performance later, as shown in the present study.

The predictive value of physiological indices for performance in military selection courses and training has been tested in several studies. Of these, physiological indices appear to have a high predictive value in the selection phases requiring high-intensity fitness. This value decreases during the training phases that require learning new skills (Sharp et al., 2012). In a recent study, Ledford and others (Ledford et al., 2020) showed that a combination of psychological (resilience questionnaire) and physiological (hormonal measures) better-predicted performance in SOF training than the use of each measure separately. As expected, in the present study, HRV differentiated between successful and unsuccessful candidates when they were tested for a short and intense time. To the best of our knowledge, this work is the first to use HRV to measure success in the Israeli SOF navy selection. Since vagal activity represents physiological and cognitive abilities (Thayer et al., 2012), a measure of HRV may differentiate between successful and unsuccessful candidates even at more advanced training stages, which require both abilities.

How could HRV predict performance in such conditions? The selection and evaluation processes examine the physiological and psychological indicators of the candidate’s resilience, assuming that these will reflect the candidate’s ability to perform in future stress conditions (Ledford et al., 2020). The neurovisceral theory assumes that the positive relationship between vagal activity and performance will be preserved even in stressful situations so that people with high resting HRV will show stable performance in challenging conditions (Hansen et al.,^2009^; Thayer et al., 2009). In the present study, psychological tests (suitability for combat service, intelligence test) were not significant predictors of success in the selection phase. Since the participants perform the psychological and personality screening as early pre-screening long before the selection test, it could be assumed that mainly the physiological indicators may differentiate between passing and failing candidates. In a recent systematic review, Tomes and his colleagues (Tomes et al., 2020) suggested using HRV to monitor populations that work in a tactical environment. The authors point out that these personnel work in a high-stress environment, and monitoring the ANS can help monitor workloads and recovery capabilities.

In the present study, we propose using HRV in the screening phase after psychological screening and other physiological indicators to identify the candidates who have the physiological resilience to cope with stressors. Given the high cost of training elite soldiers and the burden they undergo, more precise selection procedures are needed to prevent errors in recruitment. Therefore, measuring HRV may be an objective and evidence based way for identifying soldiers who may have the physiological resilience to cope with stressors, since it predicts pass or fail in a high stressful selection test. The timing of the HRV measurement is of critical importance. If HRV were to be used for selection purposes, it must be measured in a state of low or no stress, out of the context of the selection procedures.

The current study has several limitations. First, it was a retrospective study, thus, we had no control over the measurement conditions of HRV. Second, the HRV measurements were taken from ultra-short ECG (10 s). Therefore, only HRV time domain indices were taken. Similar to the other studies mentioned above, the HRV measurement was taken only at one-time point. It should be noted that in the present study, this time point was before the selection test, so the actual performance in the entire training was not examined. Along with the need for indices that will help improve the screening processes in SOF and the potential of HRV, follow-up studies are needed to understand the predictive value of HRV in the various stages of training and selection. Keeping in mind the limitations of a one-time ultra-short measure of HRV, the results of this study support the predictive value of HRV in military performance, possibly because it reflects adaptive psychological and physiological dimensions.

## Data Availability

No datasets were generated or analysed during the current study.
